# Ethical Issues around Death and Withdrawal of Life Support in Neonatal Intensive Care

**DOI:** 10.1007/s12098-021-03810-9

**Published:** 2021-06-18

**Authors:** Stuti Pant

**Affiliations:** Centre for Perinatal Neuroscience, Department of Brain Sciences, Imperial College London, London, UK

**Keywords:** Newborn, Mortality, Ethics, Bereavement, Neonatal intensive care unit (NICU), Decision-making

## Abstract

Amongst all the traumatic experiences in a human life, death of child is considered the most painful, and has profound and lasting impact on the life of parents. The experience is even more complex when the death occurs within a neonatal intensive care unit, particularly in situations where there have been conflicts associated with decisions regarding the redirection of life-sustaining treatments. In the absence of national guidelines and legal backing, clinicians are faced with a dilemma of whether to prolong life-sustaining therapy even in the most brain-injured infants or allow a discharge against medical advice. Societal customs, vagaries, and lack of bereavement support further complicate the experience for parents belonging to lower socio-economic classes. The present review explores the ethical dilemmas around neonatal death faced by professionals in India, and suggests some ways forward.

## Background

Ethical dilemmas and disagreements between health care providers and parents with regard to life-sustaining therapies are not uncommon in a neonatal intensive care environment. In the early days of neonatology, characterized by thriving medical innovation, there was an atmosphere of vibrant optimism among professionals in neonatal medicine worldwide. Advances in modern medicine and medical technologies helped in bringing down the neonatal mortality globally [1]. However, wide inequities in child mortality continue to exist; a child in born in a South Asian country has nine times more likelihood of dying in the first month of life than a child in a high-income country [2]. Over two-third of infant deaths in low and middle income countries (LMICs) still occur during neonatal period [3]. Furthermore, increased survival with advances in neonatal intensive care, may occur at the cost of major neurodisability in the survivors, particularly in the context of an extremely premature infant or an infant with severe neonatal encephalopathy. This juxtaposition of advancements and their long-term impact on critically ill newborns have unfolded complex medical, social, moral and ethical dilemmas. In addition to the tremendous cost of neonatal intensive care, there is also the risk and guilt of introducing permanently disabled children in communities with poor financial status, no social security, and lack of support services.

Ethical decisions in neonatal care are guided by Hippocratic oath and principles of beneficence, nonmaleficence, parental autonomy, correct medical facts and justice [4]. However, the complexities and uncertainties surrounding the neonatal care often make these clinical decisions ethically challenging. In LMICs such as India, these challenges are more pronounced due to significant resource constraints, including infrastructure, equipment, and staff [5, 6]. Clinicians, bound by the legal framework that do not allow for withdrawal of life support, are often forced to take precarious decisions amidst such medical uncertainties. Protecting the best interest of the baby against that of the family and state can present as an ethical challenge, especially, in situations where long-term hospital stay results in high out-of-pocket expenditure and financial hardships for the families [7].

The present review examines the development of ethical paradigm in neonatal intensive care in the global context and explores the ethical dilemmas faced by professionals in India.

## Clinical Decisions in Neonatal Intensive Care: Whose Best Interest?

In 1973, global attention was brought to ethical dilemmas relating to death and withdrawal of life support in neonatal intensive care with a groundbreaking article by Duff and Campbell [8]. Since then, this issue has attracted significant attention with regard to neonatal bioethics, roles of doctors as primary caregivers, and a shift to shared decision-making between parents and professionals. Decision-making in such fragile situations is multifaceted and often dependent on trust, relationships, life experiences, subjective interpretations of outcome, risk appetite and other personal factors, and therefore, is not simplistic in nature [9]. Complex clinical situations in neonatology are guided by the framework of principles of respect for autonomy, nonmaleficence, beneficence, and justice developed by Beauchamp and Childress in 1977, also known as ‘*Principlism*’ [10]. Nonetheless, the ethical debates in neonatology have broadly concerned the limits of decision-making around death, relative risk of survival, best interest of the child, sanctity of life, parental autonomy and decision-making, and also fair distribution of medical resources [11, 12]. Some have argued that it is in the best interest of child to limit the risk of disability as much as possible, as a poor quality of life may be worse than death; whereas others argue that by limiting advanced intensive care treatments, one may be denying a chance at life to some potential normal babies [13]. Moreover, the concept of ‘best interest’ is highly debated as the notion of it may differ between professionals and parents and have contentions of paternalistic attitude of medicine, religious, and cultural beliefs as well as individual preferences [14, 15].

Ethics and decision-making in neonatal end of life decisions has seen a paradigmatic shift from ‘doctors as information providers’ to a ‘shared decision-making’ approach in the intensive care environment [16]. As information providers, doctors viewed their responsibility in providing parents detailed information on treatment choices and potential outcomes, thereby allowing parental autonomy to decide the extent and aggressiveness of treatment for their newborn infant [17]. Complexities in this approach caused by varied levels of communication due to lack of objectivity and biases among professionals were soon appreciated. Factors such as doctor’s personal preference towards end of life as against a poor quality life of a disabled child had an influence on their assessment and communication with parents regarding potential treatments and outcomes [5]. On the other hand, there were beliefs that disagreements and conflicts occurred between doctors and parents because parents wanted treatments that were deemed inappropriate or futile by doctors [18]. This gap between the views of professionals and parents was soon realised and seen as a starting point for developing a collaborative space between the two sides. This lead to a shift from a result-focused to a process-oriented approach of shared decision-making in neonatology. This was also influenced by the research in behavioral sciences to facilitate participation and empower parental decision-making in the early 2000s [19, 20]. Professionals were now supposed to work with parents and help them recognise their own values as they faced unanticipated life-altering situations [16, 21]. As shared decision-making became an ethically preferred approach, there were questions raised about the grey zone of ethical ambiguity [22] because allowing parental preferences in decision-making was arguably not a favoured approach of professionals [23]. Some argued that since parents are not qualified to understand the clinical choices, they should be protected emotionally and not involved in the decision making, while others argued that it is the parents who have to live with the child and they must have a say in the decision-making.

In the Indian context, Professor Meharban Singh, the forefather of neonatal intensive care in India, quite rightly argued that the best interest of the baby approach may not be appropriate in LMIC settings as the baby’s interests are closely entangled with the best interest of the family. Hence, clinicians need to carefully consider the family’s circumstances and parental wishes in any decision about life-sustaining therapies [24].

With more complexities introduced in this process, it was realized that there is no simple method to communicate facts as all human communication are clouded by personal beliefs, experiences and interpretation. While professionals try to abide by their oath of doing no harm and protecting the best interests of the child and that of the family, they are bounded by vagaries of society and systemic realities. Such complexities further the need to examine the ethical issues, dilemmas and burdens of such decisions on affected parties.

## Premature Birth: Deciding the Viability of the Newborn

In high-income countries, limit of viability is usually considered as 24 wk. Extremely premature babies (born at less than 28 wk) account for the biggest group of infants in any tertiary neonatal unit in these settings. Although survival of these infants have considerably improved in the past several decades with an anticipation that advanced neonatal intensive care would reduce neurodisabilities, this has not been the case. Elegant long term follow up studies into later childhood show that a substantial proportion of the extremely premature infants have significant neurodisability and this has not improved [25]. While no reliable outcome data are available from LMICs, it is unlikely that the long term neurodevelopmental outcomes of extremely premature infants in these settings are anything but abysmal.

In India, there is a wide variation in clinical resources and intensive care facilities across public and private sector hospitals, with latter being cost prohibitive for vast majority of the population. Although individual survival stories of extremely premature infants are not infrequent, without systematic follow-up data, such stories may provide false reassurance and hope to parents. Operating under severe resource constraints, neonatal intensive care unit (NICU) professionals often apply unstandardized criteria to make decisions on allocating treatments and deciding the viability of the premature infants on a case to case basis [5, 26, 27]. Professionals also feel a responsibility to avoid burdening the family by introducing permanently disabled children into already poor communities. Moreover, in a country like India where more than 80% of health expenses are out-of-pocket expenditure, the total costs of treatment and medication for a premature infant are a substantial burden on most families [28]. Lack of systems for long term follow up and lack of longitudinal data may falsely reassure clinicians, who may be more focussed on survival than an ‘intact survival’.

## Withholding and Withdrawal of Life Support

In case of babies with severe brain injury that is likely to lead on to severe neurodisability, for example a premature infant born at 24 wk or an infant with severe encephalopathy, deciding what is best for the baby is difficult. Should these infants survive, substantial disability is likely to occur. As discussed earlier in the paper, international debates on ethical decision-making highlight the need for shared decision-making between parents and professionals. The latest consensus statement by Indian Academy of paediatrics on ‘End of Life Care’ reiterates the right to life and specifies that ‘do not resuscitate’ should not be activated till consensus is achieved between the treating team and family members, and if the family members want to include a family physician or a prominent person from the community then it should be encouraged [29]. However, in critical situations where the treatment outcomes are unclear, the ability of parents to take an informed decision in these settings is questionable. Besides, in cases where parents do not agree with the clinical judgement, it leads to ethically challenging situations where professionals are forced to take the best call, depending on existing clinical guidelines and their personal experience as well as judgement.

In Indian scenario, economic constraints of the family where long term hospital stay results in high out-of-pocket expenditure and financial hardships for the families [7] becomes a crucial deciding factor in such cases. And therefore, whether to allow a chance at life to the child or consider the poor quality of life the child may have, is a common dilemma faced by professionals. Irrespective of parents understanding and these challenging decisions, it is the ethical responsibility of the treatment team to explain different aspects of baby’s condition, short term and long term complications and possible neurodevelopmental disabilities and help them make the right decision.

## The ‘Male Child’ or a ‘Precious Child’ Concept

Preference to a male child or a precious child are not just alien concepts in high-income countries, but are also illegal, as every child is equally important. However, within the Indian context, pregnancy and child care practices are shaped by socio-cultural beliefs, and this often has a bearing on parents’ attitudes and perceptions about critical care of their sick newborns. Apparent gender preferences among parents are seen, especially in rural areas and decisions taken about the NICU care is often influenced by such preferences. For instance, parents often go up to the extent of exhausting their financial resources to save male babies, as boys are viewed as economic assets, dowry earners and also the support system for parents in their old age. Similarly, in case of precious pregnancies or firstborn, parents often continue the NICU care, irrespective of their financial status.

One of the most significant and commonly faced ethical issues is gender based discrimination and sex selection. While gender differences during postneonatal period have been called out as a major threat to survival of female infants [30], it is also prevalent within the neonatal intensive care environment. Professionals actively try and forbid gender based discrimination in such decisions. However, often in order to avoid the risk of neonatal abandonment in the hospitals [31], they find themselves giving into parental demands. Generally, a family’s decision to withdraw care from their newborn or for leaving against medical advice is cost based, but there have been evidence to report gender-based motivation [5]. As against the perks of having a male heir to the family, a female child is still considered a financial burden in many communities in the country due to many discriminatory customs that are still practiced. Furthermore, mothers are likely to receive the blame for the sex of the child and for the increased costs, and such stigmatising conditions can heighten the risk of home eviction for both, the mother and her child [31]. Such extremities and awareness of these realities add to the ethical dilemmas experienced by professionals, where they battle to ensure nondiscrimination in decisions and also protect the best interests of the child, and, in some cases, the mother.

## Why National Guidelines are Required?

It is acknowledged that ethically challenging scenarios in newborn care cannot have a uniform approach and that they need to be reviewed on a case to case basis, guidelines by national academic bodies may provide a framework for clinician. In the UK, The Royal College of Paediatrics and Child Health (RCPCH) published the first guidelines on ‘Withholding or Withdrawing Life Saving Treatment in Children: A Framework for Practice’ in 1997 following extensive consultations. This framework was expected to be a living document and was revised in 2004, and subsequently in 2015. It acknowledges that all members of the health team, in partnership with parents, have a duty to act in the best interests of the child, which includes sustaining life and restoring health to an acceptable standard. However, there are circumstances in which treatments that merely sustain ‘life’ neither restore health nor confer other benefits and hence, are no longer in the child’s best interests. Therefore, the framework recommends situations where withdrawal of life-sustaining treatments may be appropriate when life is limited in quantity (i.e. brain stem death, inevitable or imminent death) or in quality (i.e. when it is difficult or impossible for the child to derive benefit from continued life) [32]. In vast majority of cases, this framework has enabled professionals and parents to work together, and facilitating decisions about withdrawing life-sustaining therapy to be taken in partnership with parents.

Unlike in the UK, withdrawal of life support in the Indian neonatal intensive care do not have any legal backing. Often in these situations, due to accumulating costs parents may decide to discharge the baby against medical advice. Such decisions further increase the complexity and the trauma experienced by the family, when the baby deteriorates in front of the parents and subsequently dies on-route or at home. With so support available, parents are likely to succumb to severe mental health breakdowns and familial complications. The experience is more discriminatory and shattering in nature towards both mothers and families belonging to low socio-economic backgrounds.

Currently there is no standard national guidelines in India which specifies the period of viability or indications for ‘do not resuscitate’. India Newborn Action Plan, which was developed in 2014 as a response to the Global Every Newborn Action Plan (ENAP), with an objective of accelerating the reduction of preventable newborn deaths and stillbirths in the country aims to attain a single digit neonatal mortality and stillbirth rate by 2030 [33]. This plan also details the required care of healthy newborn, care of small and sick newborn and care beyond newborn survival as key pillars. However, it still does not address any ethical dilemmas commonly observed in NICUs, specifically in issues related to newborn death discussed in this paper.

Until recently, such communication and ethical issues had not been a part of medical curriculum or neonatal training in India. In a positive step forward, the National Medical Council of India introduced the module on medical ethics and communication as part of the undergraduate curriculum. Inclusion of appropriate training in ethical principles, practical ethics, reasoning, communication and reflection is necessary in order to prepare, empower and sensitise neonatologists to effectively approach and address the many ethical dilemmas they encounter on a day-to-day basis. While this move was long-awaited, it is important to realise that classroom teaching alone may not be sufficient to address these ethical issues and complex dilemmas. The discussions need to start at an even earlier stage starting from admission to medical schools in India. For instance, the Medical Schools Council in the UK requires the admission process to assess the ability and suitability of applicants for medicine as a ‘caring profession’ and often applying students aim to obtain prior experience with voluntary sector in health or related areas to understand the needs of this profession [34]. Interpersonal skills such as communication and empathy are considered critical requirements of medicine as a profession and are deeply embedded as part of the training. With the complexities in the Indian socio-cultural and economic setting, it is very essential that medical education in India implements a well-rounded training programme in ethics and communication.

Death of a child is one of the most traumatic and painful experience that parents have to go through. Last few decades have seen an increase in acknowledgement of impact of perinatal losses and bereavement for parents and families in high-income countries [35]. However, despite high rates of child mortality, the public health system in LMICs such as India does not acknowledge the impact of child deaths on parents and families. There is an urgent need for a multi-level bereavement and grief support system in these hospitals, starting at the hospital level. An active role must be played by the NICU doctors and nurses, and community level support must be extended from medium to long term to services such as family counseling, bereavement helpline, professionally guided parental support groups.

## Conclusion

The current state of research on ethical issues experienced in neonatal intensive care in India is inadequate and fragmented. There is an urgent need for development of national level policy and framework on selection and management of babies needing NICU care, with clear guidelines on gestational age, birth weight, medical complications and decisions to withhold or withdraw care. Learning from the models applied to the high-income countries, more research is needed to develop models that are relevant to the local socio-cultural context and support professionals on how to cope with dilemmas in a resource-constrained setting. Most importantly, it is crucial to acknowledge the importance of ethical dilemmas, decisions, and conflicts by incorporating ethics as part of countrywide medical education programme. Responsibility of professionals including both doctors and nurses is not only limited to the neonate’s time spent in intensive care, but also extends to support the family in navigating grief and life in the community. It has become imperative to identify best practices, devise evidence-based guidelines, and train professionals in bereavement and palliative care to provide quality support to the family.
